# Minimal alveolar concentration of sevoflurane in combination with remimazolam in adults during laryngeal mask insertion: an up-down sequential allocation study

**DOI:** 10.1186/s12871-024-02468-y

**Published:** 2024-03-08

**Authors:** Yan Huang, HongYu Liao, LinJi Li, Juan Xu, PingPing Jiang, YanXia Guo, KunPeng Liu

**Affiliations:** 1 Department of Anesthesiology, The Second Clinical Medical College, North Sichuan Medical College, Nanchong Central Hospital, Nanchong, China; 2 Department of Anesthesiology, Affiliated Hospital of North Sichuan Medical College, Nanchong, Sichuan China; 3 Department of Anesthesiology, Peking University International Hospital, Beijing, China

**Keywords:** Remimazolam, Sevoflurane, Laryngeal mask airway, Minimum alveolar concentration

## Abstract

**Background:**

Remimazolam is a novel ultrashort-acting intravenous benzodiazepine sedative-hypnotic. The combination of remimazolam and sevoflurane does not increase respiratory sensitivity, produce bronchospasm, or cause other adverse conditions. We aimed to observe the effects of different remimazolam doses on the minimum alveolar concentration (MAC) of sevoflurane at end-expiration during laryngeal mask insertion and evaluate the effect of sex on the efficacy of the combination of remimazolam on the suppression of laryngeal mask insertion in adult patients.

**Methods:**

We included 240 patients undergoing laparoscopic surgery under general anesthesia with elective placement of a laryngeal mask (120 males and 120 females). The patients were randomly divided into four groups according to sex: a control group (randomization for female patients, RF0; randomization for male patients, RM0) and three remimazolam groups (RF1, RM1 / RM2, RF2 / RM3, RF3), with 30 patients in each group. Induction was established by vital capacity rapid inhalation induction (VCRII), using 8% sevoflurane and 100% oxygen (6 L/min) in all patients. The (RF1, RM1), (RM2, RF2), and (RM3, RF3) groups were continuously injected with remimazolam at doses of 1, 1.5, and 2.0 mg/kg/h, respectively, while the (RM0, RF0) group was injected with an equal volume of normal saline. The end-expiratory concentration of sevoflurane was adjusted to a preset value after the patient’s eyelash reflex disappeared. After the end-expiratory concentration of sevoflurane was kept stable for at least 15 min, the laryngeal mask was placed, and the patient’s physical response to the mask placement was observed immediately and within 30 s of placement. The MAC of sevoflurane was measured using the up-and-down sequential method of Dixon.

**Results:**

The calculated MAC of end-expiratory sevoflurane during laryngeal mask insertion in adult females was (2.94 ± 0.18)%, (2.69 ± 0.16)%, (2.32 ± 0.16)% and (1.83 ± 0.15)% in groups RF0, RF1, RF2 and RF3; (2.98 ± 0.18)%, (2.80 ± 0.19)%, (2.54 ± 0.15)% and (2.15 ± 0.15)% in male groups RM0, RM1, RM2 and RM3, respectively. The MAC values were significantly lower in the (RF1-RF3, RM1-RM3) group when compared to the (RF0, RM0) group. There was no significant difference between (RF0, RF1) and (RM0, RM1), but the MAC value of the RF2-RF3 group was significantly lower than that of the RM2-RM3 group.

**Conclusions:**

Remimazolam can effectively reduce end-expiratory sevoflurane MAC values during laryngeal mask placement in adults. When remimazolam was measured above 1.5 mg/kg/h, the effect of inhibiting laryngeal mask implantation in female patients was stronger than that in male patients. Remimazolam at a dose of 1–2 mg/kg/h combined with sevoflurane induction can be safely and effectively used in these patients.

## Introduction

The laryngeal mask airway (LMA) is a commonly used device for airway management during short surgical procedures due to its simplicity and fewer complications than tracheal intubation [1–3]. Sevoflurane is a commonly used drug for inhalation induction of anesthesia but using it solely for induction can lead to adverse effects such as respiratory and circulatory depression, hypotension, and brain wave changes [4]. Combining drugs during induction can improve the efficiency and safety of anesthesia. Remimazolam is a new benzodiazepine that is fast-acting, metabolizes rapidly, and does not rely on hepatic function, with sex differences in drug metabolism [5]. It has minimal circulatory and respiratory depression when continuously administered intravenously and does not increase respiratory sensitivity or produce bronchospasm when combined with sevoflurane [6–9]. The objective of this study is to examine the effect of different doses of remimazolam on end-expiratory sevoflurane MAC values during laryngeal mask placement stimulation in adults, with the goal of providing clinical guidance on the safe and rational use of this drug and to evaluate the effect of sex on the efficacy of the combination of remimazolam on the suppression of laryngeal mask insertion in patients.

## Methods

### Ethics approval and study design

The study was approved by the Ethics Committee of Affiliated Hospital of North Sichuan Medical College, Nanchong, China (Approval No. 2018ER (R) 048) and was registered in the center of the Chinese Clinical Trials Registry at http://www.chictr.org.cn (ChiCTR2300075498). Written informed consent was obtained from all participants before enrollment. A total of 240 patients expected to undergo laparoscopy under general anesthesia with laryngeal mask placement, ASAI-II class, aged 20–45 years, were selected and randomly divided into four groups according to sex differences of 30 expected cases each. Inclusion criteria: ASA I-II class, age 20–45 years, body mass index (BMI) 18–30 kg/m^2^, no history of upper respiratory tract and pulmonary infections 2 weeks prior to surgery, no history of asthma, and estimated operative time within 2 hours. Exclusion criteria: patients with suspected difficult airway, obesity, history of gastroesophageal reflux, history of congenital heart disease, allergy to benzodiazepines and sevoflurane, family history of malignant hyperthermia. A random number table method was used to randomly divide 240 patients who met the inclusion criteria into 1 control group (RF0, RM0) and 3 remimazolam groups (RF1, RM1 / RM2, RF2 / RM3, RF3) according to sex differences. Withdrawal criteria: patients with regurgitant misaspiration during the trial; patients with hypoxemia and laryngospasm during the trial; patients with laryngeal mask placement time greater than 30 s; patients with a heart rate < 50 bpm requiring pharmacological management; patients who cannot maintain stability with sevoflurane at end-expiration.

### Anesthesia administration

All patients routinely abstained from drinking and eating before surgery, and no preoperative medication was given. After admission, vital signs were monitored using a multifunctional monitor: noninvasive blood pressure, electrocardiogram, pulse oxygen saturation, body temperature, partial pressure of end-expiratory carbon dioxide, and bispectral index (BIS). Peripheral vein access was established, and 10 ml/kg of compound sodium chloride was injected. During the induction of anesthesia, all patients were induced by vital capacity rapid inhalation induction (VCRII). As required by VCRII, the patients were asked to first take a deep breath, followed by maximum exhalation. Afterwards, the anesthetic circuit with facemask was applied, and the patients were asked to take a deep breath, hold their breath for as long as possible, and then breathe out to the residual volume. All patients were using 8% sevoflurane (Shanghai Hengrui Pharmaceutical Co., Ltd., lot no. H20070172) and 100% oxygen (6 L/min) by inhalation, while remimazolam (Yichang Renfu Pharmaceutical Co., Ltd., lot no. 171,301–201,801) was continuously pumped intravenously in the (RF1, RM1), (RM2, RF2), and (RM3, RF3) groups at 1 mg/kg/h, 1.5 mg/kg/h, and 2.0 mg/kg/h, respectively, while the (RF0, RM0) group was pumped with an equal volume of saline. When the patient’s eyelash reflex disappears, adjust the inhaled oxygen concentration (2 L/min) and sevoflurane end-expiratory concentration to the preset values. Sevoflurane inhalation induction may be assisted by ventilation to achieve a pulse oximetry saturation greater than 95%. The end-expiratory concentration of sevoflurane was kept stable for at least 15 min before the mask placement, and the patients’ somatic responses to the mask were observed immediately and within 30 s after the mask placement. The determination of mask placement and somatic reaction was done by the same experienced attending anesthesiologist in all patients, and the effect was determined based on the patient’s response to the laryngeal mask stimulation. Prior to placement, lidocaine paste was applied to the surface of the laryngeal mask in all patients with the aim of lubricating it and alleviating discomfort after removal. To prevent damage caused by repeated operations, this procedure should be performed by experienced attending physicians. CLMA-type mask model selection criteria: According to the mask instruction manual specification selection reference table, 3.0 for weight 30–50 kg and 4.0 for weight 50–70 kg.

### Determination of MAC

The MAC of sevoflurane combined with different doses of remimazolam for each group was determined by using an up-and-down sequential allocation technique.

Classified responses by the patient to CLMA insertion as either ‘movement’ or ‘no movement’. Movement was defined as the presence of bucking, straining, laryngospasm, or gross purposeful muscular movement within 30 s of airway insertion.

Based on the pretest results, the sevoflurane inhalation concentrations were 3.01%, 2.79%, 2.22%, and 2.03% for the first patient in each female group, and 3.11%, 2.83%, 2.74%, and 2.35% for the first patient in each male group, respectively. If there was a positive or negative response at the time of mask placement, the end-expiratory concentration of sevoflurane in the next patient was increased or decreased by 0.2% from that of the previous patient until six positive to negative or negative to positive crossover points were observed in each group of patients. The MAC value of sevoflurane for this group of patients was determined by taking the average of the six crossover points [10, 11]. After successful placement of the laryngeal mask, the remimazolam pumping was stopped, and intravenous anesthetics were given promptly. Ventilator-controlled ventilation with a tidal volume of 6–8 ml/kg, a respiratory rate of 14–16 breaths/min, and an end-expiratory CO_2_ maintained at 35–45 mmHg. If the patient suffered laryngospasm or difficulty in mask placement, the patient was switched to intravenous anesthesia, ciatracurium was given as a muscle relaxant, and endotracheal intubation was substituted for mask placement while the patient was withdrawn from the trial. After observation of all patients, routine static inhalation of compound general anesthesia was performed, and the appropriate depth of anesthesia was adjusted so that BIS monitoring was maintained between 40 and 60 to complete the procedure [12]. After the operation, the patient was sent to the recovery room, and the mask was removed after full consciousness. Patients were followed up postoperatively for the occurrence of painful pharyngeal discomfort and intraoperative awareness after mask removal.

### Secondary data collection

Mean arterial pressure (MAP) and heart rate (HR) were monitored at the following time points: just before saline/remimazolam administration (baseline values), before and after 1 min of laryngeal mask placement. BIS were recorded before, at 5 and 10 min after remimazolam pumping, and before and 1 min after laryngeal mask placement.

### Statistical analysis

The sample size was calculated using G*Power analysis (version 3.1.9.7, Germany), with an alpha of 0.05 and a power of 0.80. Each group was expected to require 30 patients, considering the dropout rate [13].

SPSS 23.0 statistical software was used for statistical analyses. Continuous measurement data following a normal distribution were characterized by their mean and standard deviation (mean ± SD). The data pertaining to ASA grade were presented in numerical form, and a χ2 test was employed to compare differences between the groups. The MAC was determined using the up-and-down method, which involved calculating the mean end-tidal sevoflurane concentration at the six crossing points (excluding non-moving to moving points). The preoperative data (age, BMI, basal MAP, and basal HR) and intraoperative data (BIS, MAP, and HR) were compared among the groups using one-way analysis of variance (ANOVA). Differences in vital signs at different time points were assessed using a repeated-measures ANOVA. All statistical tests were conducted as two-sided, and a P-value of less than 0.05 was deemed to be statistically significant.

## Results

**Fig. 1:**
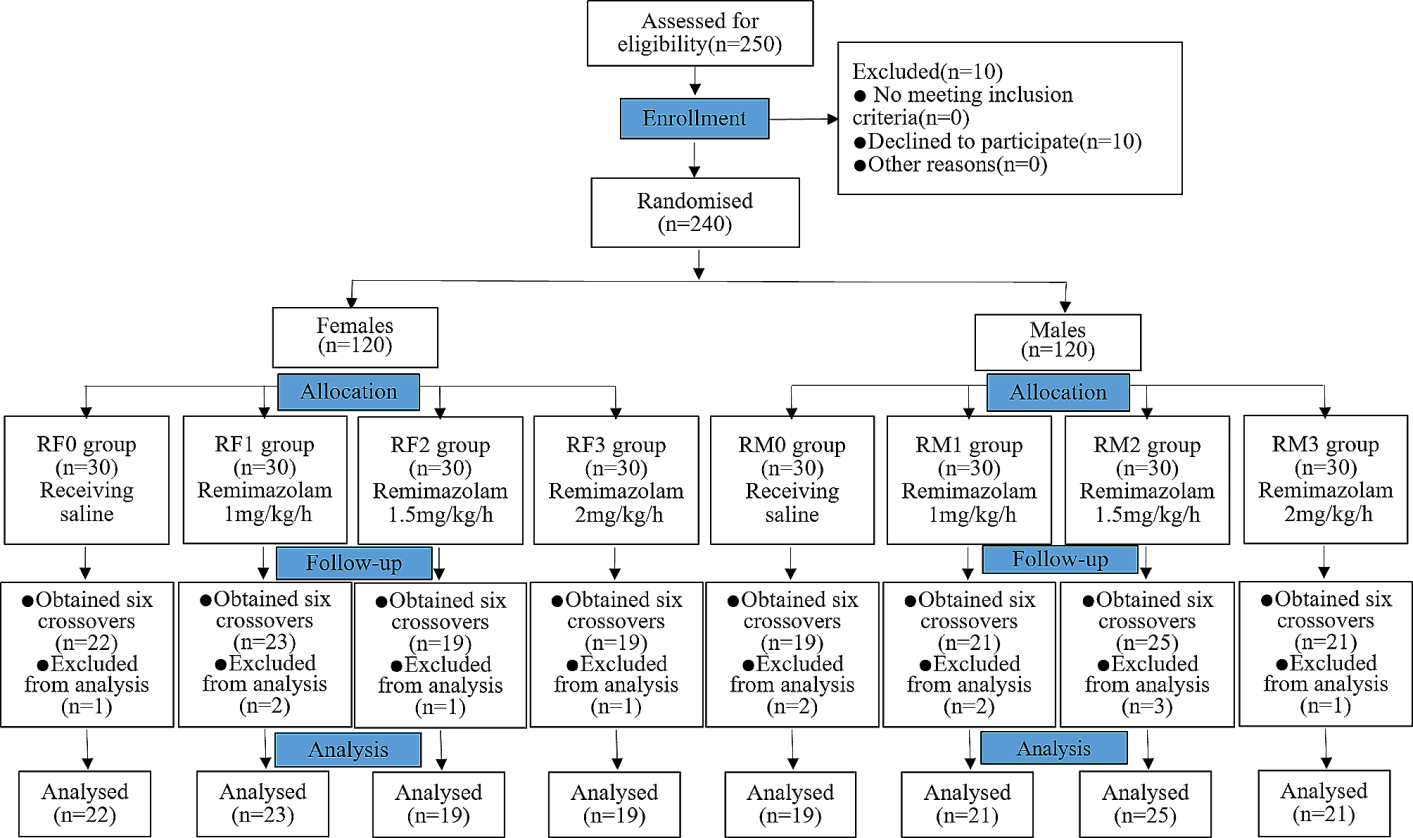
Flow chart of the patient selection and analysis process for the inclusion in the study

Out of the 240 patients that were expected, 182 participated in the trial. According to experimental observation, in each of the four groups, RF0, RF1, RF2, RF3, RM0, RM1, RM2, and RM3 had 1, 2, 1, 1, 2, 2, 3, 1 patients withdraw from the trial because the end-expiratory sevoflurane concentration could not be maintained stable. Finally, according to sex differences, in each of the four groups, RF0, RF1, RF2, and RF3 obtained six crossover points from positive to negative or from negative to positive, and the number of cases used was 22, 23, 19, and 19; RM0, RM1, RM2, and RM3 obtained six crossover points from positive to negative or from negative to positive, and the number of cases used was 19, 21, 25, and 21, respectively (Fig. 1).

The general information of each group according to the sex differences of patients is shown in Tables 1 and 2. The differences in age, BMI, basal MAP, HR, and ASA classification of the respective patients were not statistically significant, excluding the interference of baseline data such as age as a confounding factor (Tables 1 and 2).

**Table 1:** Comparison of general information of the four groups of female patients

Index	Group RF0	Group RF1	Group RF2	Group RF3	*P* value
Age (year)	32.8 ± 7.7	32.0 ± 6.8	33.1 ± 7.0	32.1 ± 7.7	0.95
BMI (kg/m²)	23.8 ± 4.2	22.4 ± 3.6	25.0 ± 3.3	24.1 ± 3.6	0.12
Basal MAP (mmHg)	94.1 ± 6.1	94.2 ± 6.3	96.1 ± 5.2	95.1 ± 5.5	0.63
Basal HR (bpm)	96.9 ± 10.6	97.6 ± 10.6	98.1 ± 9.5	98.0 ± 10.5	0.98
ASA class (I/II)	9/13	12/11	9/10	13/6	0.348

Data are presented as the mean ± standard deviation (SD) or as appropriate, as the number of patients. BMI, Body mass index; MAP, mean arterial pressure; HR, heart rate; ASA, American Society of Anesthesiologists

**Table 2:** Comparison of general information of the four groups of male patients

Index	Group RM0	Group RM1	Group RM2	Group RM3	*P* value
Age (year)	31.8 ± 7.6	32.7 ± 6.9	34.0 ± 6.2	31.5 ± 7.6	0.62
BMI (kg/m²)	23.7 ± 3.4	24.1 ± 2.9	25.4 ± 2.3	24.1 ± 3.3	0.24
Basal MAP (mmHg)	93.2 ± 3.6	93.5 ± 3.8	93.4 ± 4.7	94.6 ± 3.0	0.68
Basal HR (bpm)	96.8 ± 6.2	96.9 ± 5.4	98.3 ± 5.8	96.9 ± 5.4	0.76
ASA class(I/II)	10/9	8/13	11/14	13/8	0.44

Data are presented as the mean ± standard deviation (SD) or as appropriate, as the number of patients. BMI, body mass index; ASA, American Society of Anesthesiologists, HR, heart rate; MAP, mean arterial pressure

### Primary outcome

**Fig. 2:**
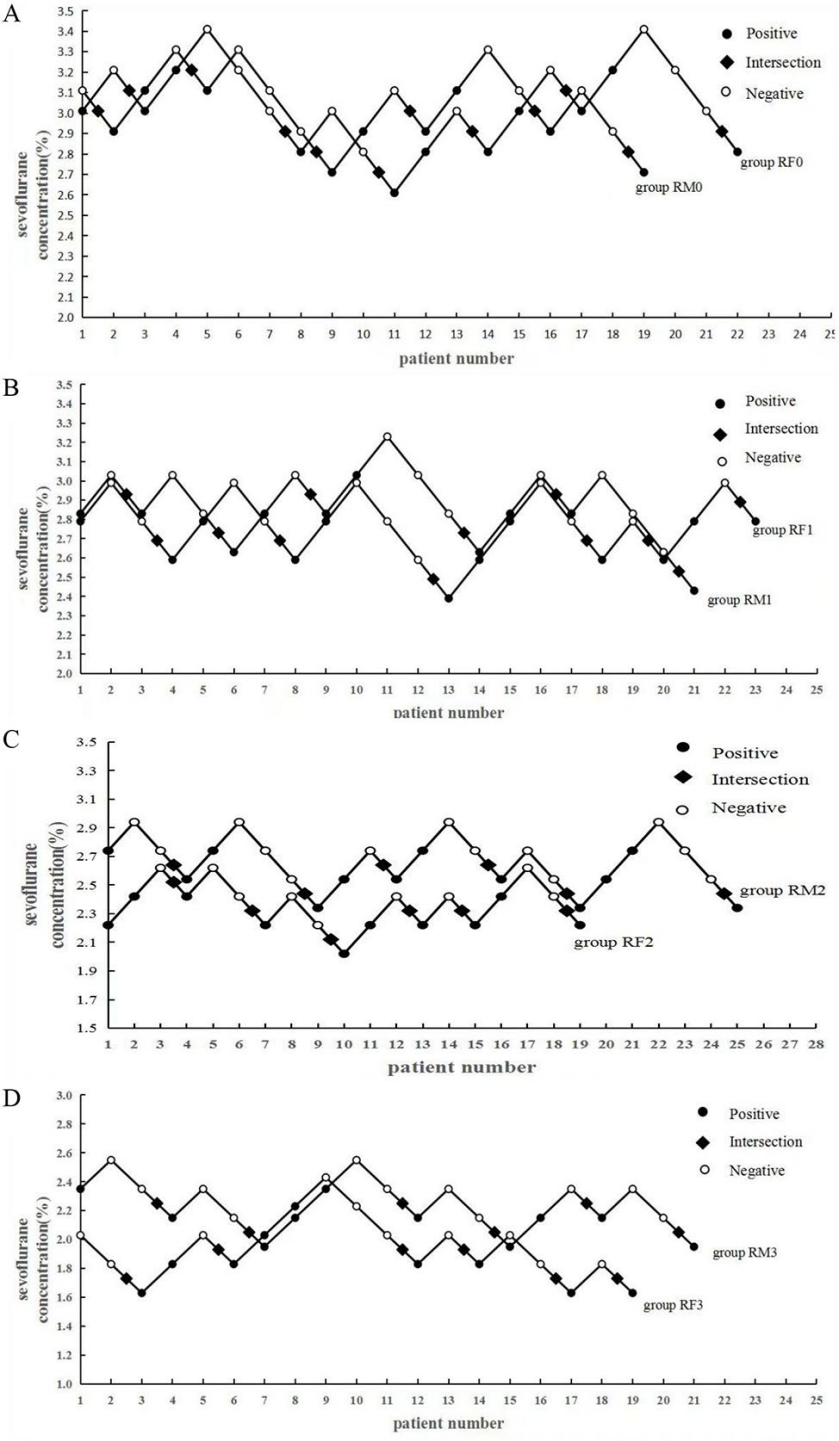
MAC values of sevoflurane in each group of patients. (**A**) MAC values of sevoflurane in (RF0, RM0) group. (**B**) MAC values of sevoflurane in (RF1, RM1) group (**C**) MAC values of sevoflurane in (RF2, RM2) group (**D**) MAC values of sevoflurane in (RF3, RM3) group

The sequential concentrations of sevoflurane combined with remimazolam using the up-and-down method are demonstrated. The MAC of sevoflurane was (2.94 ± 0.18)%, (2.69 ± 0.16)%, (2.32 ± 0.16)%, and (1.83 ± 0.15)% for the dose groups of RF0, RF1, RF2, and RF3, respectively, and decreased by approximately 8.5%, 21%, and 38% in the RF1-RF3 groups compared with the RF0 group (Fig. 1). The EC95 (95% CI) values of sevoflurane for the laryngeal mask placement, using the centered isotonic regression analysis, were (2.83–3.06)%, (2.59–2.79)%, (2.22–2.42)%, and (1.74–1.92)% in the RF0, RF1, RF2, and RF3 groups, respectively (Fig. 2; Table 3). The MAC of sevoflurane was (2.98 ± 0.18)%, (2.80 ± 0.19)%, (2.54 ± 0.15)%, and (2.15 ± 0.15)% for the dose groups of RM0, RM1, RM2, and RM3, respectively, and decreased by approximately 6%, 15%, and 28% in the RM1-RM3 groups compared with the RM0 group (Fig. 1). The EC95 (95% CI) values of sevoflurane for the laryngeal mask placement, using the centered isotonic regression analysis, were (2.86–3.09)%, (2.68–2.92)%, (2.45–2.63)%, and (2.06–2.24)% in the RM0, RM1, RM2, and RM3 groups, respectively (Fig. 2; Table 4). In the comparison between different sex groups, there was no significant difference between (RF0, RF1) and (RM0, RM1), but the MAC value of the RF2-RF3 groups was significantly lower than that of the RM2-RM3 groups (Table 5).

**Table 3:** Sevoflurane MAC and its 95% CI for the four female groups

Group	RF0	RF1	RF2	RF3	*P* value
MAC (%)	2.94	2.69	2.32	1.83	0.001
95% CI (%)	2.83–3.06	2.59–2.79	2.22–2.42	1.74–1.92	--

**Table 4:** Sevoflurane MAC and its 95% CI for the four male groups

Group	RM0	RM1	RM2	RM3	*P* value
MAC (%)	2.98	2.80	2.54	2.15	0.001
95% CI (%)	2.86–3.09	2.68–2.92	2.45–2.63	2.06–2.24	--

**Table 5:** Comparison of MAC values between male and female groups at the same dose

	R0	R1	R2	R3
Female groups	2.94	2.69	2.32	1.83
Male groups	2.98	2.80	2.54	2.15
*P* value	0.64	0.15	0.02	< 0.001

MAC, minimum alveolar concentration; CI, confidence intervals

Non-movements are marked with ^0^, and movements are marked with •. The doses of remimazolam in the RF0, RF1, RF2, and RF3 groups were 0, 1.0, 1.5, and 2.0 mg/kg/h, respectively. To get six crossovers, 22, 23, 19, and 19 patients were included individually in groups RF0, RF1, RF2, and RF3. The doses of remimazolam in the RM0, RM1, RM2, and RM3 groups were 0, 1.0, 1.5, and 2.0 mg/kg/h, respectively. To get six crossovers, 19, 21, 25, and 21 patients were included individually in the RM0, RM1, RM2, and RM3 groups.

### Secondary outcome

Changes in MAP and HR before and after laryngeal mask placement in each group of patients (Table 6). There was no statistically significant difference in MAP and HR values before mask placement in each group, and MAP and HR were significantly higher after mask placement than before mask placement, with statistically significant differences.

**Table 6:** Changes in mean MAP and HR before the placement of laryngeal mask in each group

	MAP (mmHg)				HR (bpm)			
Group	Before placement	After placement	Z	*P* value	Before placement	After placement	Z	*P* value
RF0	78.1 ± 6.5	85.5 ± 6.8*	2.50	< 0.01	72.9 ± 9.8	87.2 ± 10.5*	4.11	< 0.01
RM0	78.2 ± 4.9	86.5 ± 6.0*	3.74	< 0.01	71.9 ± 5.7	86.1 ± 9.1*	4.12	< 0.01
RF1	78.1 ± 7.1	84.6 ± 8.2*	4.02	< 0.01	74.8 ± 10.0	83.6 ± 11.4*	3.77	< 0.01
RM1	79.2 ± 5.3	87.4 ± 6.2*	3.71	< 0.01	72.9 ± 5.8	85.2 ± 7.9*	4.42	< 0.01
RF2	78.9 ± 6.9	88.1 ± 7.5*	3.62	< 0.01	73.3 ± 7.9	82.7 ± 10.9*	3.82	< 0.01
RM2	79.6 ± 4.4	86.1 ± 5.3*	3.9	< 0.01	72.4 ± 6.8	81.2 ± 8.4*	3.46	< 0.01
RF3	76.6 ± 7.1	85.5 ± 7.9*	3.73	< 0.01	73.0 ± 10.0	83.7 ± 15.4*	3.23	< 0.01
RM3	78.4 ± 5.3	85.2 ± 6.0*	3.3	< 0.01	72.2 ± 6.2	83.9 ± 7.7*	4.16	< 0.01

Data are presented as the mean standard deviation (SD)

HR, heart rate; MAP, mean arterial pressure

*P < 0.05 vs. before CLMA placement

**Fig. 3:**
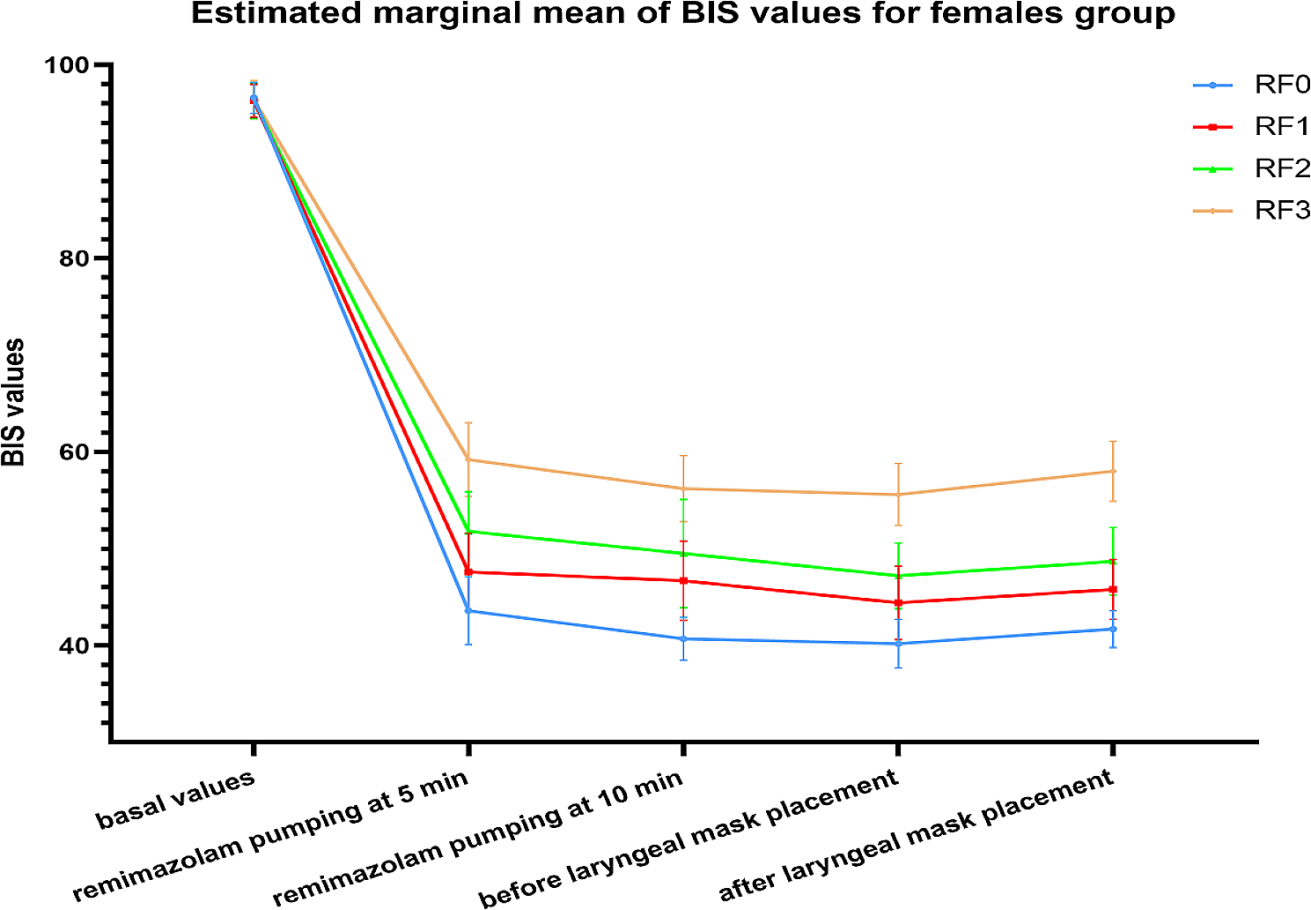
Changes in BIS values of patients in the female group

**Fig. 4:**
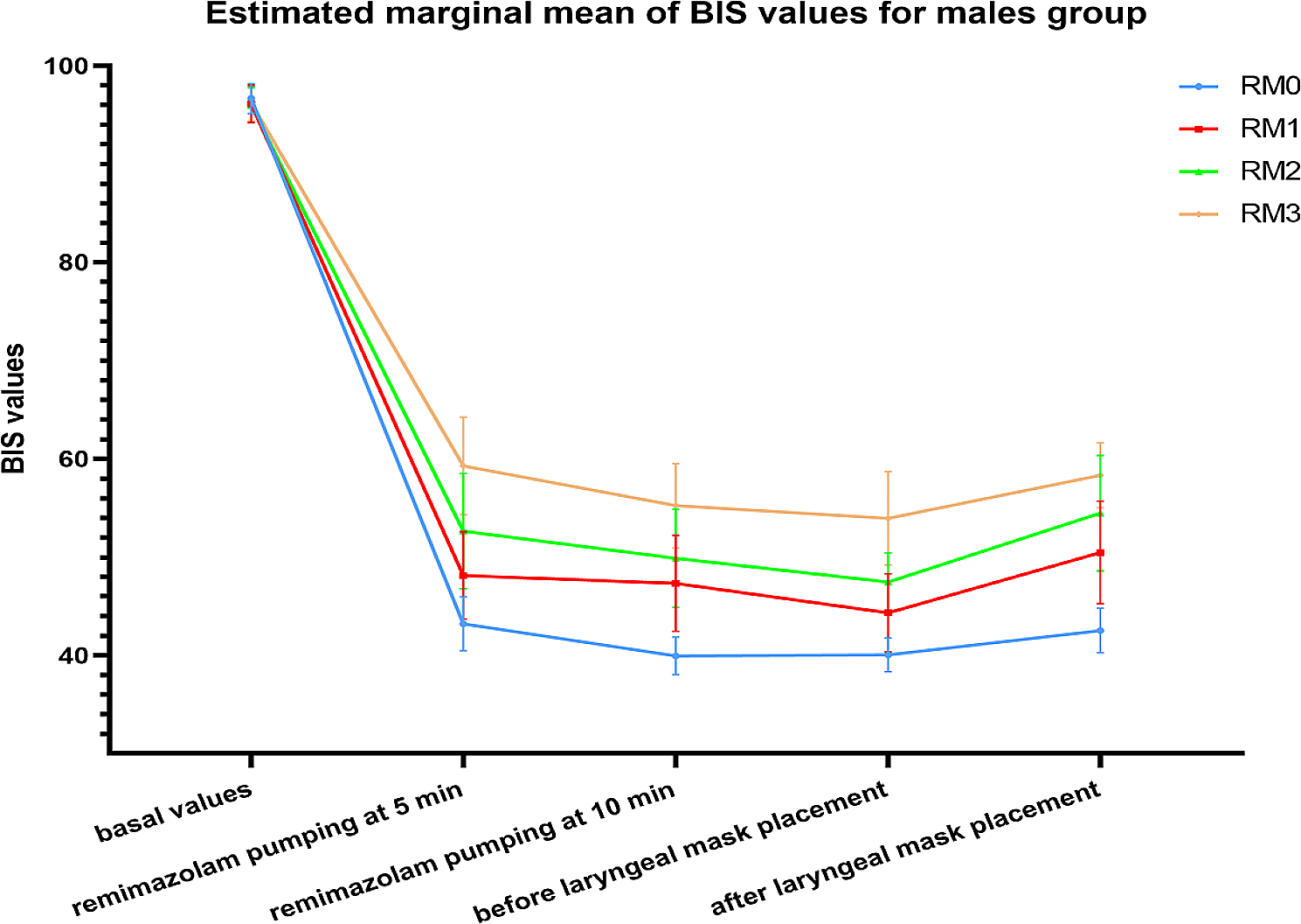
Changes in BIS values of patients in the male group

Changes in BIS values at different moments in each group of patients. When compared between groups at the same time point, there was no difference in BIS values between each of the four groups with different sexes before remimazolam pumping; compared with the (RF0, RM0) group, the BIS values increased in the (RF1-RF3, RM1-RM3) group before and 1 min after remimazolam pumping at 5 min, 10 min, and laryngeal mask placement. BIS values gradually increased with increasing remimazolam doses, and the difference was statistically significant. When compared between groups at different time points, the BIS values were reduced in the remimazolam pumping 5 min, 10 min, and 1 min before and after laryngeal mask placement in the (RF0-RF3, RM0-RM3) group compared to the basal values, with statistically significant differences. There was no difference in BIS values between the (RF0-RF3, RM0-RM3) groups when comparing before and 1 min after the laryngeal mask placement. There was no significant difference between the different sex comparison groups at the corresponding time points (Figs. 3 and 4).

## Discussion

The study measured the MAC of sevoflurane in adult patients aged 20–45 years undergoing laryngeal mask placement. The MAC value of sevoflurane without remimazolam was found to be (2.94 ± 0.18)% in female patients and (2.98 ± 0.18)% in male patients, similar to those found in previous studies [14]. However, Zaballos et al. used a sevoflurane concentration adjustment gradient of 0.5% and kept the end-expiratory concentration of sevoflurane stable for 10 min, while the concentration adjustment gradient used in this experiment was 0.2% and the end-expiratory concentration of sevoflurane was stable for at least 15 min. A smaller concentration adjustment gradient and stable end-expiratory gas concentration could improve the accuracy of MAC determination. Therefore, the results measured in this trial should be relatively more accurate. Compared to the previous study, the age range of patients selected for this trial was different and the type of laryngeal mask used was different, which also contributed to some extent to the difference in MAC values. In our study, when inhaling sevoflurane alone, there was no difference in MAC between females and males; MAC did not correlate with sex. However, when remimazolam was measured above 1.5 mg/kg/h, the effect of inhibiting laryngeal mask implantation in female patients was stronger than that in male patients [15]. The study also strengthened patient temperature monitoring and management to ensure that patients’ body temperature was maintained within the normal range and excluded patients with underlying diseases such as cardiovascular diseases [14]. Patients who could not maintain stable end-expiratory sevoflurane concentrations were excluded from the trial.

Laryngeal mask placement required lower end-expiratory sevoflurane concentrations than endotracheal intubation [16], but patients still needed to achieve a certain depth of anesthesia to ensure smooth hemodynamics during mask placement. Remimazolam was used in this study to reduce the MAC values of sevoflurane during inhalation induction of anesthesia. The study found that remimazolam reduced the MAC values of sevoflurane by 8.5%, 21%, and 38% at doses of 1 mg/kg/h, 1.5 mg/kg/h, and 2.0 mg/kg/h in females and 6%, 15%, and 28% at doses of 1 mg/kg/h, 1.5 mg/kg/h, and 2.0 mg/kg/h in males, respectively. Therefore, when anesthesia is induced with remimazolam combined with sevoflurane, the inhalation concentration of sevoflurane can be appropriately reduced. When the dose of remimazolam is greater than 1.5 mg/kg/h, female patients may choose a lower inhalation concentration of sevoflurane compared to male patients to avoid excessive anesthesia.

The BIS values gradually increased with increasing doses of remimazolam, probably due to the decrease in sevoflurane at increasing doses of remimazolam and the relatively small effect of remimazolam under anesthesia on patients’ BIS and status index [17, 18]. The BIS values for each group were significantly reduced and were in sedation at different time points after inhalation induction. There was no significant difference in BIS values before and after laryngeal mask placement, indicating that the end-expiratory concentration of sevoflurane was selected reasonably. No intraoperative awareness occurred in any of the patients at the postoperative visit.

The limitations of this study include not analyzing other adverse effects and only following up on the occurrence of intraoperative awareness after the operation. There may also be some subjective factors in the determination of laryngeal mask placement, related to the operator’s clinical experience. The combined application in different age groups needs further study. In conclusion, continuous pumping of remimazolam during induction of sevoflurane anesthesia has a significant sedative effect and also reduces the MAC value of sevoflurane during inhalation induction of anesthesia. When the dose of remimazolam was greater than 1.5 mg/kg/h, the effect on female patients was significantly better than that of male patients.

## Data Availability

The datasets used and/or analysed during the current study are available from the corresponding author on reasonable request.

## Abbreviations

ASA: American Society of Anesthesiologists
BIS: Bispectral index
cLMA: Classic laryngeal mask airway
HR: Heart rate
LMA: Laryngeal mask airway
MAC: Minimum alveolar concentration
MAP: Mean arterial pressure
VCRII: Vital capacity rapid inhalation induction
